# Contribution of the region Glu181 to Val200 of the extracellular loop of the human P2X1 receptor to agonist binding and gating revealed using cysteine scanning mutagenesis^1^

**DOI:** 10.1111/j.1471-4159.2009.06035.x

**Published:** 2009-05

**Authors:** Jonathan A Roberts, Marie Valente, Rebecca C Allsopp, David Watt, Richard J Evans

**Affiliations:** Department of Cell Physiology & Pharmacology, University of LeicesterLeicester, UK

**Keywords:** agonist, ATP, binding, mutagenesis, P2X receptor

## Abstract

At the majority of mutants in the region Glu181-Val200 incorporating a conserved AsnPheThrΦΦxLys motif cysteine substitution had no effect on sensitivity to ATP, partial agonists, or methanethiosulfonate (MTS) compounds. For the F185C mutant the efficacy of partial agonists was reduced by ∼ 90% but there was no effect on ATP potency or the actions of MTS reagents. At T186C, F188C and K190C mutants ATP potency and partial agonists responses were reduced. The ATP sensitivity of the K190C mutant was rescued towards WT levels by positively charged (2-aminoethyl)methanethiosulfonate hydrobromide and reduced by negatively charged sodium (2-sulfonatoethyl) methanethiosulfonate. Both MTS reagents decreased ATP potency at the T186C mutant, and abolished responses at the F195C mutant. ^32^P-2-azido ATP binding to the mutants T186C and K190C was sensitive to MTS reagents consistent with an effect on binding, however binding at F195C was unaffected indicating an effect on gating. The accessibility of the introduced cysteines was probed with (2-aminoethyl)methanethiosulfonate hydrobromide-biotin, this showed that the region Thr186-Ser192 is likely to form a beta sheet and that accessibility is blocked by ATP. Taken together these results suggest that Thr186, Phe188 and Lys190 are involved in ATP binding to the receptor and Phe185 and Phe195 contribute to agonist evoked conformational changes.

The ATP sensitive P2X receptor family comprises seven receptor subunits (P2X1–7) that assemble as homo- or hetero-trimeric ion channels (North and Surprenant 2000). They are a distinct class of ligand gated cation channels with two transmembrane segments, intracellular amino and carboxy termini and a large extracellular loop (Roberts *et al.* 2006). ATP is released into the extracellular space in a variety of ways including vesicular release from neurons, in response to shear stress, and following cell damage. P2X receptors are involved in a range of processes including neuronal excitability, pain sensation and bone formation (Burnstock 2006). A consensus of key amino acids involved in ATP action is emerging (Evans 2009). Positively charged lysine residues 68, 70 and 309 (P2X1 receptor numbering) seem likely to be involved in co-ordinating the binding of the negatively charged phosphates of ATP (Ennion *et al.* 2000; Jiang *et al.* 2000; Zemkova *et al.* 2007), and in addition Lys308 in the P2X2 receptor (Lys309 P2X1 receptor numbering) may contribute to channel gating (Cao *et al.* 2007). The AsnPheArg (290–292 P2X1 numbering) motif has been suggested to be involved in agonist binding and gating (Roberts and Evans 2007; Evans 2009). For P2X1 receptors an inter-subunit disulphide bond can form between cysteine mutants K68C and F291C suggesting their close proximity in the agonist binding site (Marquez-Klaka *et al.* 2007) and a similar finding has been reported for P2X2 receptors, in the case of the equivalent mutations in the P2X3 and P2X4 receptors the distance between the residues is slightly larger (Marquez-Klaka *et al.* 2009).

A conserved region in the middle of the extracellular loop incorporating a phenylalanine-threonine doublet (Phe185Thr186 P2X1 receptor numbering) was also identified in P2X1 receptors that when mutated resulted in decreased ATP potency and partial agonist action (Roberts and Evans 2004, 2006). Recent studies on cysteine mutants of the P2X2 and P2X4 receptors suggest that ATP responses at the conserved threonine, but not the phenylalanine of the doublet, can be modified by methanethiosulfonate (MTS) reagents (Roberts *et al.* 2008). Mutation of the nearby conserved lysine to alanine (Lys190 in P2X1) resulted in an ∼ 5, 200 and 2000-fold decrease in ATP potency at P2X1, P2X2 and P2X4 receptors respectively (Ennion *et al.* 2000; Jiang *et al.* 2000; Yan *et al.* 2005) suggesting this may also contribute to agonist action. This region of the receptor contains a high degree of amino acid conservation and for human receptors in the twenty amino acid stretch Glu181-Val200 (P2X1 receptor numbering) five of them are totally conserved and a further five show a conservative substitution including the AsnPheThrΦΦxLys motif (Fig. 1). Bioinformatic analysis predicts that part of this region (Thr186-Ser192), incorporating residues that when mutated have been shown to modify ATP sensitivity, forms a beta sheet (Digby *et al.* 2005). Non-conserved residues also play important roles in mediating the differences between the properties of the P2X subunits and may have a regulatory role in this region. However a systematic analysis of the role of this region of the receptor, that could substantiate the structural predictions, has not been undertaken to date.

**Fig. 1:**
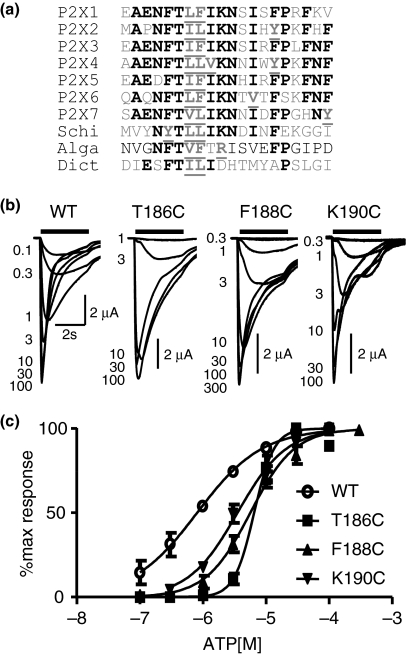
Effects of cysteine point mutants of E181 to V200C on ATP potency at human P2X1 receptors. (a) Sequence line-up for the region corresponding to E181 to V200 of the P2X1 receptor for the seven human, *Schistosoma mansoni* (Schi) (Agboh *et al.* 2004), the green alga *Ostreococcus tauri* (Alga) (Fountain *et al.* 2008) and amoeba *Dictyostelium discoidium* (Dict) (Fountain *et al.* 2007) P2X receptors. Residues conserved in at least five of the human isoforms are shown in black bold, conservative substitutions are shown underlined in bold gray. (b) Concentration responses to ATP for oocytes expressing WT, T186C, F188C and K190C mutant P2X1 receptors. Application of ATP is indicated by bar. (c) Summary of concentration response data for WT and mutants T186C, F188C and K190C that showed significant decreases in ATP potency (*n* = 3–4).

The substituted cysteine accessibility method has been useful in probing the extracellular regions close to the transmembrane segments and their contribution to ATP action at the P2X receptor (Jiang *et al.* 2000; Roberts and Evans 2007). In this study we have used cysteine substitution mutagenesis of the region Glu181-Val200 of the P2X1 receptor to (i) determine the effects of cysteine substitution on ATP sensitivity and the efficacy of partial agonists, (ii) establish whether charged cysteine reactive MTS reagents modify ATP responses, (iii) address whether mutations and MTS compounds result in an effect on agonist binding or channel gating using a 2-azido ATP binding assay, and (iv) utilized an (2-aminoethyl)methanethiosulfonate hydrobromide (MTSEA)-biotinylation assay to measure the accessibility of introduced cysteine residues, and whether this is sensitive to receptor activation. These studies provide a novel insight into the structural organization and properties of this region of the receptor.

## Methods

### Site-directed mutagenesis

Cysteine point mutations for residues Glu181–Val200 were introduced via the QuikChange™ mutagenesis kit (Stratagene, La Jolla, CA, USA) using a human P2X1 receptor plasmid as the template as described previously (Ennion *et al.* 2000). Production of the correct mutations and absence of coding errors in the P2X1 mutant constructs was verified by DNA sequencing (Automated ABI Sequencing Service, University of Leicester).

### Expression in *Xenopus laevis* oocytes

Wild type and mutant constructs were transcribed to produce sense strand cRNA (mMessage mMachine™, Ambion, Austin, TX, USA) as described previously (Ennion *et al.* 2000). Manually defolliculated stage V *Xenopus laevis* oocytes were injected with 50 nL (50 ng) of cRNA using an Inject + Matic microinjector (J. Alejandro Gaby, Genéva, Switzerland) and stored at 18°C in ND96 buffer (96 mM NaCl, 2 mM KCl, 1.8 mM CaCl_2_, 1 mM MgCl_2_, 5 mM sodium pyruvate, 5 mM HEPES, pH 7.6). Media was changed daily prior to recording 3–7 days later.

### Electrophysiological recordings

Two-electrode voltage clamp recordings (at a holding potential of −60 mV) were carried out on cRNA injected oocytes using a GeneClamp 500B amplifier with a Digidata 1322 analog-to-digital converter and pClamp 8.2 acquisition software (Axon Instruments, Molecular Devices, Foster City, CA, USA) as previously described (Ennion *et al.* 2000). Native oocyte calcium activated chloride currents in response to P2X receptor stimulation were reduced by replacing 1.8 mM CaCl_2_ with 1.8 mM BaCl_2_ in the ND96 bath solution. ATP (Mg salt) was applied via a U-tube perfusion system as was 2′,3′-*O*-(4-benzoyl)-ATP (BzATP) and P^1^,P^5^-diadenosine 5′-pentaphosphate (Ap_5_A) (all from Sigma, Poole, UK). ATP was applied at 5-min intervals. Using this regime reproducible ATP evoked responses were recorded. Individual normalized concentration response curves were fitted with the Hill equation: *Y*= [(*X*)^H^/[(*X*)^H^ + (EC_50_)^H^] where *Y* is response, *X* is agonist concentration, H is the Hill coefficient, and EC_50_ is the concentration of agonist evoking 50% of the maximum response. pEC_50_ is the –log_10_ of the EC_50_ value. For the calculation of EC_50_s individual concentration response curves were generated for each experiment and statistical analysis carried out on the pEC_50_ data generated. In the figures concentration response curves are fitted to the mean normalized data.

### Characterisation of the effects of methanethiosulfonate compounds

To study the effect of MTS compounds on ATP activation at wild type and cysteine mutants, ATP (∼ EC_50_ concentration) was applied and either MTSEA or sodium (2-sulfonatoethyl) methanethiosulfonate (MTSES) (Toronto Research Chemicals, Toronto, Canada) were bath-perfused (for at least 5 min; the recovery time required between application to see reproducible responses) prior to application of ATP via the U-tube. MTS reagents (1 mM) were prepared immediately before use. The effect of MTS reagents on the concentration responses to ATP were investigated following 1-h incubation with MTSEA (1 mM) or 3-h incubation with MTSES (1 mM) to irreversibly modify all the P2X receptors, following washout of MTS reagents ATP was applied via the U-tube with ND96 bathing solution (no MTS reagents present). We have previously shown that concentration response curves generated with MTS reagents in the bath or after longer term treatment followed by washout of the MTS reagents give the same results for a range of P2X1 receptor cysteine mutants (Roberts and Evans 2007).

### ^32^P-2-azido ATP radiolabelling of the P2X1 receptor

To assess any effects of MTS reagents on agonist binding we used a ^32^P-2-azido ATP binding assay as described previously (Roberts *et al.* 2008). We used this for wild type (WT), T186C, K190C and F195C mutants. Densitometry analysis was carried out on the resultant films with data corrected by background subtraction calculated for each lane and expressed as percentage of control for WT or a particular mutant.

### Western blotting

(2-Aminoethyl)methanethiosulfonate hydrobromide biotin was used to probe the various cysteine mutants to map the extracellular region E181-V200 of the P2X1 receptor as described previously (Roberts *et al.* 2008). MTSEA biotin reacts covalently with sulfhydryl groups provided by accessible cysteine residues. The wild type P2X1 receptor contains no ‘free’ cysteine residues (Ennion and Evans 2002) and therefore an introduced cysteine would be free to bind MTS compounds if accessible.

### Analysis

All data are shown as mean ± standard error of the mean. Significant differences between WT and mutants were calculated by one way analysis of variance followed by Dunnett’s test for comparisons of individual mutants against control using Graphpad Prism 5 (GraphPad Software Inc., San Diego, CA, USA). The significance of any changes in ATP potency by MTS compounds, and the effects of ATP on MTSEA-biotinylation at particular mutants were determined with the appropriate Student’s *t*-test. *n* corresponds to the number of oocytes tested for electrophysiological data, and for biochemical studies experiments were repeated at least three times.

## Results

### Effects of cysteine substitution on agonist action

At WT P2X1 receptors ATP evoked concentration dependent desensitizing inward currents with an EC_50_ of ∼ 0.8 μM as reported previously (Roberts and Evans 2005). There were no significant differences in the amplitude of peak inward currents evoked by a maximal concentration of ATP between WT and any of the mutants E181C to V200C (Table 1). For the majority of the mutants in addition there was no effect on ATP sensitivity. This confirms previous studies on alanine mutants of residues Glu183, Asn184, Asn191, Pro196 and Phe198 (Jiang *et al.* 2000; Ennion *et al.* 2001; Roberts and Evans 2004, 2005, 2006). The P2X1 receptor F185C mutant had no effect on ATP potency similar to an alanine mutation at the equivalent position in the Dictyostelium P2X receptor (Fountain *et al.* 2007), but contrasts with a previous P2X1 receptor mutant F185A that had a 10-fold decrease in potency (Roberts and Evans 2004), and the 3 and 20-fold decreases in potency for the equivalent mutants at P2X2 and P2X4 receptors (Zemkova *et al.* 2007; Roberts *et al.* 2008). There were significant decreases in ATP EC_50_ to 6.5, 5.8 and 3.4 μM for the mutants T186C, F188C and K190C respectively and also a slowing in the time-course of desensitization of the current (823 ± 160, 1720 ± 233, 2392 ± 246 and 1720 ± 213 ms for the 50% decay time for the ATP evoked current for WT and mutants respectively, *p* < 0.001 for the mutants compared to WT, *n* = 7–11, Fig. 1). This pattern of alternative residues showing a decrease in ATP potency is consistent with the beta sheet prediction for this region of the receptor (Digby *et al.* 2005) (that is substantiated by the biotinylation studies – see later) that could face the ATP binding pocket. In addition the Hill slope of the T186C mutant was significantly increased almost threefold to 2.95 ± 0.07 (Table 1).

**Table 1:** Effects of cysteine substitution on peak response and ATP action

	Peak current (nA)	ATP pEC_50_	Hill
WT	9725 ± 621	6.10 ± 0.07	1.01 ± 0.05
E181C	9509 ± 1014	5.94 ± 0.17	0.73 ± 0.05
A182C	11 874 ± 853	5.75 ± 0.11	1.25 ± 0.31
E183C	11 422 ± 1030	5.67 ± 0.23	0.88 ± 0.20
N184C	10 729 ± 900	6.18 ± 0.11	0.88 ± 0.04
F185C	12 561 ± 1506	5.76 ± 0.13	1.33 ± 0.06
T186C	8065 ± 1286	5.19 ± 0.06***	2.95 ± 0.07***
L187C	11 730 ± 877	5.76 ± 0.12	1.11 ± 0.08
F188C	9799 ± 662	5.25 ± 0.09***	1.26 ± 0.06
I189C	10 578 ± 655	5.81 ± 0.19	0.86 ± 0.07
K190C	8411 ± 745	5.45 ± 0.08*	1.15 ± 0.03
N191C	7048 ± 538	6.01 ± 0.03	1.04 ± 0.08
S192C	9553 ± 519	5.88 ± 0.10	0.92 ± 0.06
I193C	7573 ± 663	6.00 ± 0.14	1.02 ± 0.10
S194C	9159 ± 813	6.21 ± 0.06	0.93 ± 0.13
F195C	7551 ± 647	6.14 ± 0.15	0.82 ± 0.10
P196C	10 056 ± 704	6.02 ± 0.07	1.14 ± 0.05
R197C	8268 ± 1081	5.96 ± 0.13	1.04 ± 0.20
F198C	8586 ± 816	6.10 ± 0.24	0.85 ± 0.12
K199C	8926 ± 825	6.10 ± 0.12	1.03 ± 0.16
V200C	10 426 ± 1004	5.99 ± 0.09	0.87 ± 0.86

Peak current values taken on the first application of a maximal concentration of ATP (100 μM). pEC_50_ values shown as calculated from fits of individual concentration response curves. pEC_50_ is –log10 of the EC_50_ for ATP. Hill coefficient of fitted curves is shown. Significant differences from WT P2X1 receptors are indicated *p < 0.01 and ***p < 0.001, *n* = 8–27 for peak currents, *n* = 3–4 for pEC_50_.

**Fig. 2:**
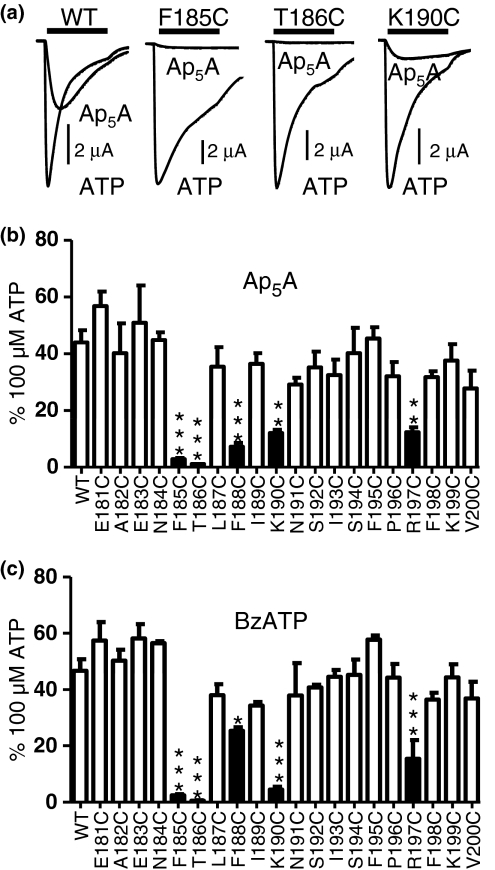
Efficacy of the partial agonists Ap_5_A and BzATP. (a) Currents evoked by ATP and Ap_5_A application (both 100 μM) at WT, F185C, T186C and K190C mutant P2X1 receptors. Agonist application for 3 s is indicated by bar. (b) Summary of the efficacy of the partial agonist Ap_5_A (100 μM) expressed as a percentage of the response to a maximal concentration of ATP (100 μM). (c) Summary of the efficacy of the partial agonist BzATP (100 μM) expressed as a percentage of the response to a maximal concentration of ATP (100 μM). Significant reductions in efficacy are shown as black bars, *p < 0.05, **p < 0.01, ***p < 0.001, (*n* = 3–5).

At WT receptors the partial agonists Ap_5_A and BzATP have an efficacy of ∼0.45–0.50 as described previously (Roberts and Evans 2004). At the majority of mutants, like for sensitivity to ATP, there was no change in the efficacy of the partial agonists (Fig. 2). For two mutants, F185C and R197C that had no change in ATP potency the partial agonist responses were reduced, at F185C both BzATP and Ap_5_A efficacy was 0.03 and at R197C responses were reduced to ∼ 0.13 for the two agonists. At the mutants T186C, F188C and K190C, with a decrease in ATP potency there was also a significant decrease in the efficacy of the partial agonists (Fig. 2), this was greatest for the T186C mutant (efficacy < 0.01). At WT P2X1 receptors the pEC_50_ for Ap_5_A was 6.2 ± 0.5, this was unaffected for R197C (5.92 ± 0.23, *p* = 0.23) but sensitivity to Ap_5_A was reduced for both F188C and K190C mutants (pEC_50_ 5.33 ± 0.16 and 5.70 ± 0.07 respectively, *p* < 0.001 for both, *n* = 3) (the responses were too small to study partial agonists sensitivity at F185C and T186C mutants). The sensitivity to BzATP was also reduced compared to WT (pEC_50_ 6.52 ± 0.1) for the mutants F188C and R197C (pEC_50_s 5.40 ± 0.06 and 5.52 ± 0.01 respectively, *p* < 0.001 for both, *n* = 3, the responses were too small to study the partial agonist sensitivity of BzATP at K190C).

### Effects of MTS reagents at cysteine mutants

**Fig. 3:**
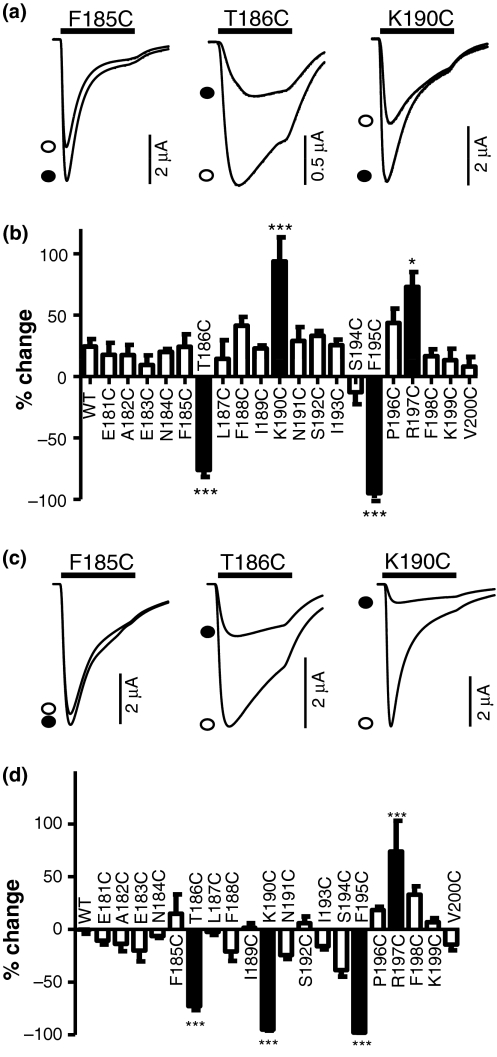
Effects of charged MTS reagents on ATP evoked P2X1 receptor currents. (a) Responses to an ∼ EC_50_ concentration of ATP are shown before (open symbol) and after the application of 1 mM MTSEA (filled symbol) for the mutants F185C, T186C and K190C. ATP application (3 s) is indicated by bar. (b) Summary of the effects of MTSEA on WT and mutant P2X1 receptors. (c) Responses to an ∼ EC_50_ concentration of ATP are shown before (open symbol) and after the application of 1 mM MTSES (filled symbol) for the mutants F185C, T186C and K190C. ATP application (3 s) is indicated by bar. (d) Summary of the effects of MTSES on WT and mutant P2X1 receptors. Data are expressed as % change (0% indicates no change), on MTS application, significant differences from WT are shown by black bars, *p < 0.05, ***p < 0.001, (*n* = 3–14).

Methanethiosulfonate reagents have been used to modify accessible cysteine residues and in investigating the site of agonist action at P2X receptors (Jiang *et al.* 2000; Roberts and Evans 2007; Roberts *et al.* 2008). Charged MTS reagents have been particularly useful as the ATP molecule has a negatively charged phosphate tail, and under physiological conditions magnesium is complexed with ATP giving rise to localized positive charge associated with the agonist. We have therefore determined whether ATP evoked responses at mutant receptors can be modified by positively charged MTSEA and negatively charged MTSES. An EC_50_ concentration of ATP was used to test the effects of MTS reagents as this would show the greatest sensitivity to any modification. Positively charged MTSEA (1 mM) potentiated the amplitude of responses at WT P2X1 receptors by 24.4 ± 6.1%, similar to that described previously for WT P2X1 receptors (Roberts and Evans 2007). For sixteen of the mutants there was no significant difference in the effects of MTSEA (Fig. 3). Responses were increased by 93.7 ± 19.6% and 73.0 ± 11.9% for K190C and R197C mutants and inhibited by 76.4 ± 4.6% and 73.0 ± 11.9% for T186C and F195C mutants respectively. The maximal effects of the MTSEA were generally observed on the first application of ATP following 5-min treatment with MTSEA.

Following washout of MTSEA WT responses returned to control values (0.5 ± 7.4% change from control). For R197C the effects of MTSEA were immediately reversed on washout (to 2 ± 6.7% change from control) this suggests that the effects of MTSEA at R197C do not result from the irreversible modification of the introduced cysteine residue. The inhibitory effects of MTSEA at T186C and F195C were still significant following 5-min washout of MTSEA however the level was reduced to 44.8 ± 7.2% and 36.2 ± 6.7% respectively. Similarly for K190C the potentiatory effect of MTSEA was maintained albeit at a reduced level following washout of MTSEA (28.6 ± 8.4% potentiation). Trafficking of new P2X1 receptors from the intracellular space that have not been modified by MTSEA could account for this partial reversal and a similar phenomenon was seen for cysteine mutants of the P2X4 receptor (Roberts *et al.* 2008). We previously showed that 1-h pre-incubation followed by washing with membrane permeant MTSEA was sufficient to modify the pool of P2X4 receptors and overcome the apparent reversibility of the effect (Roberts *et al.* 2008). Following 1-h pre-treatment with MTSEA followed by washing the peak amplitude of WT currents was unaffected (6.6 ± 8.3% change compared to control). In contrast T186C and F195C mutants were reduced by 80.5 ± 6.2% and 91.7 ± 2.2% compared to control following pre-treatment consistent with that seen with MTSEA in the bath and indicative of an irreversible modification. Responses of K190C to 1 μM ATP were potentiated by 220 ± 28% by 1-h pre-treatment with MTSEA consistent with an irreversible modification of the introduced cysteine residue. R197C mutants were unaffected by pre-treatment showing that this mutant is not irreversibly modified by MTSEA and the reversal of responses on washout of MTSEA does not result from rapid trafficking of unmodified receptors to the cell surface. These results demonstrate that P2X1 receptors are likely to undergo constitutive trafficking in oocytes and partial washout of MTSEA effects at T186C, F195C and K190C mutants is likely to result from trafficking of un-modified receptors to the cell surface. Longer incubation with MTSEA allows modification of the whole pool of P2X receptors and results in irreversible modification of T186C, F195C and K190C mutants with no effect on WT or R197C mutant receptors. This is consistent with previous studies on P2X1 receptor cysteine mutants where the effects of MTS reagents on ATP concentration response curves were the same whether the MTS was applied with ATP or in the absence of MTS reagents following long term exposure to irreversibly modify the whole pool of introduced cysteine residues (Roberts and Evans 2007).

Negatively charged MTSES (1 mM) had no effect on WT P2X1 receptor currents in response to an EC_50_ concentration of ATP similar to that reported previously (Roberts and Evans 2007). For 16 of the mutants there was no significant effect compared to WT (Fig. 3). At R197C responses were potentiated by ∼ 70% however they returned to control following washout (−6 ± 4.9%) and it seems that like the response to MTSEA results from a reversible effect on the channel. For T186C, K190C and F195C ATP evoked currents were reduced by 80–95%, and following 5-min washout of MTSES they were still inhibited significantly by 45–55%. This once again suggests that new unmodified receptors are being trafficked to the cell surface during the washout. We have previously shown that a 3-h incubation with membrane impermeant MTSES is sufficient to irreversibly modify the total pool of P2X4 receptors (Roberts *et al.* 2008). When oocytes expressing these P2X1 receptor mutants were pre-treated with MTSES for 3 h, followed by washout, a similar level of inhibition was seen as for bath application, indicating that the partial reversal results from constitutive trafficking of un-modified receptors to the cell surface (and complete turnover of the receptor pool occurs within 3 h).

### Effects of MTS reagents on ATP potency and agonist binding

**Fig. 4:**
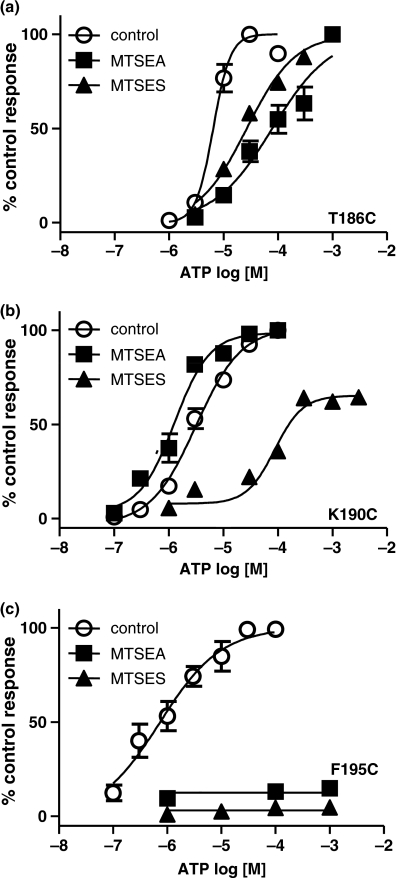
Effects of MTS reagents on ATP concentration responses at P2X1 receptor mutants. Concentration response curves to ATP were determined under control conditions or following incubation with MTSEA (1 mM for 1 h followed by washout) or MTSES (1 mM for 3 h followed by washout). (a) At T186C the EC_50_ was significantly increased by both MTSEA and MTSES with no effect on the peak response. (b) At K190C MTSEA significantly increased whilst MTSES significantly decreased ATP potency. For MTSES peak current responses were also reduced. (c) MTSEA and MTSES treatment reduced responses at the F195C mutant receptor by > 90% even for a maximal concentration of ATP (1 mM).

The mutants T186C and F195C were inhibited by both MTSEA and MTSES whereas at the K190C mutant the effects of the MTS reagents were dependent on the charge, with potentiation by positively charged MTSEA and inhibition by negatively charged MTSES. To determine whether the MTS reagents had an effect on ATP potency and/or the amplitude of maximal responses we constructed concentration response curves following MTS treatment (1 h MTSEA or 3 h MTSES followed by washing) to irreversibly modify free cysteine residues (Fig. 4). At the T186C mutant MTSEA and MTSES treatment decreased ATP potency ∼ 10 and 5-fold (*p* < 0.005) with no effect on the maximal response. In addition the Hill slope was reduced back to normal WT levels on treatment with the MTS reagents however the mechanism associated with this is unclear. At the K190C mutant ATP potency was increased ∼ 3-fold by MTSEA (*p* < 0.05) with no effect on the maximal response whilst MTSEA decreased ATP potency threefold (*p* < 0.001) and the maximal response by ∼35% (*p* < 0.01). At the F195C mutant ATP responses to 1 mM ATP were reduced by > 90% by both MTSEA and MTSES (*p* < 0.001).

**Fig. 5:**
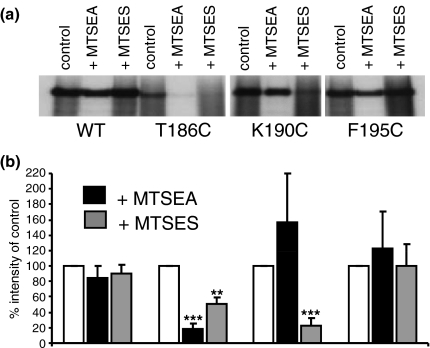
Effects of MTS reagents on ^32^P-2-azido ATP cross-linking to WT and mutant P2X1 receptors. (a) Oocytes expressing WT and mutant P2X1 receptors were UV irradiated in the presence of ^32^P-2-azido ATP and P2X1 receptors were isolated by immunoprecipitation and run on a gel and exposed to X-ray film. The autoradiographs show the level of radioactivity associated with the P2X1 receptors under control conditions and the effects of MTSEA or MTSES (both 1 mM). (b) Summary data of the effects of MTSEA and MTSES on 2-azido ATP cross-linking to WT, T186C, K190C and F195C mutant P2X1 receptors. Data are expressed as % of control condition for each group of oocytes and corrected for background levels. (*n* = 4 batches of oocytes for each). **p < 0.05, ***p < 0.001.

The effects of the MTS reagents on the concentration response curves for T186C, K190C and F195C could result from an effect on the binding of ATP to the receptor and/or the subsequent conformational change associated with gating of the ionic pore. Radiolabelled 2-azido ATP cross-linking can be used to measure binding to P2X1 receptors (Roberts and Evans 2007; Agboh *et al.* 2009). In this study, we have tested whether 2-azido ATP binding to WT and mutant P2X1 receptors is sensitive to modification by MTS reagents (Fig. 5). ^32^P-2-azido ATP cross-linking to the WT P2X1 receptor was unaffected by MTSEA or MTSES treatment consistent with the little or no effect of these compounds on ATP evoked responses. At the T186C mutant 2-azido ATP cross-linking under control conditions was reduced by 33 ± 4% compared to WT (*p* < 0.01) consistent with the decrease in ATP potency. 2-azido ATP cross-linking was further reduced by both MTSEA (*p* < 0.001) and MTSES (*p* < 0.05) and mirrored their inhibitory effects on ATP evoked currents. At K190C there was no change in 2-azido ATP binding compared to control. There was a trend towards an increase in 2-azido ATP binding consistent with the increase in ATP potency following MTSEA application however this did not reach significance. The lack of significance may reflect that small changes in ATP potency (threefold) are on the threshold of sensitivity that can be detected in the binding assay. However MTSES reduced 2-azido ATP cross-linking at K190C by ∼ 80% (*p* < 0.001) consistent with the rightward shift in agonist sensitivity and a depression of the maximal response to ATP. These effects at T186C and K190C are indicative of an effect of MTS reagents on agonist binding. For the F195C mutant MTSEA or MTSES had no effect on the cross-linking of 2-azido ATP even though these MTS reagents essentially abolished ATP evoked responses. This shows that the MTS reagents do not affect agonist binding but inhibit the gating of the F195C mutant channel. Overall these results suggest that the ^32^P-2-azido ATP cross-linking assay can be used to differentiate effects of the MTS reagents on agonist binding or the subsequent gating of the channel.

### Conformational dependent accessibility of cysteine mutants

**Fig. 6:**
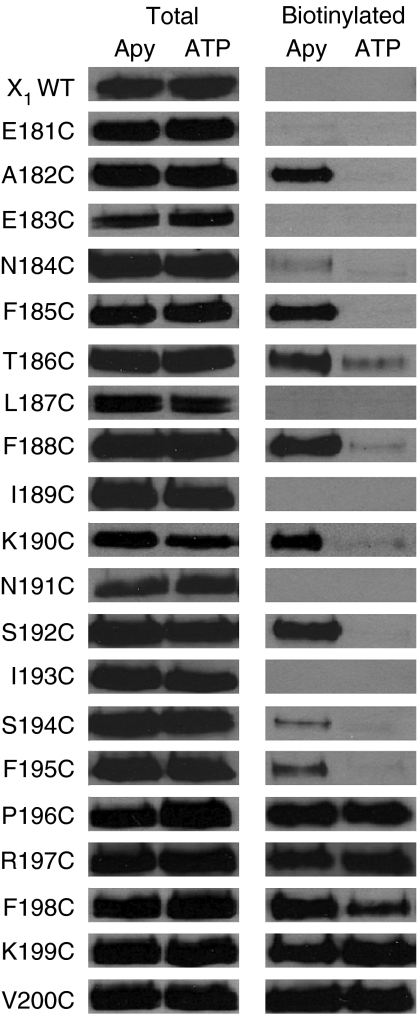
MTSEA-biotinylation reveals the surface accessibility of the region E181-V200. Representative western blots show the equivalent total levels of WT and P2X1 receptor mutants expressed in the oocytes used for the biotinylation assay (left hand panel). To determine the surface accessibility of introduced cysteine residues oocytes were incubated with MTSEA-biotin, and biotinylated proteins isolated, run on a gel and blotted with an anti-P2X1 receptor antibody. Oocytes were either pre-treated with apyrase (to break down any endogenous nucleotides) or ATP (to activate the receptor). For WT receptors no biotinylation could be detected. Biotinylation was also below the limit of detection for E181C, E183C, L187C, I189C, N191C and I193C. At N184C and S194C weak biotinylation that was ATP sensitive was recorded. ATP sensitive biotinylation was detected at A182C, F185C, T186C, F188C, K190C, S192C and F195C. MTSEA-biotin was observed for P196C-V200C mutants either in the absence of presence of ATP. Blots are representative of those from three to eight separate batches of oocytes.

Bioinformatic predictions suggest that part of Glu181-Val200 region may form a beta sheet however there is no direct evidence to verify this, or to suggest whether this portion of the receptor is accessible at the receptor surface. We have therefore used an MTSEA-biotinylation assay to map the surface accessibility of the cysteine substituted region and to determine whether it is sensitive to the activation state (Roberts and Evans 2007) (Fig. 6). No MTSEA-biotinylation was detected for WT P2X1 receptors either under resting conditions (in the presence of apyrase to break down any ATP released from the oocytes) or following ATP application as reported previously (Roberts and Evans 2007) and consistent with the ten cysteine residues in the extracellular domain forming five disulphide bonds (Ennion and Evans 2002). MTSEA-biotinylation was below the threshold for detection for the mutants E181C, E183C, L187C, I189C, N191C and I193C indicating that these introduced cysteine residues are buried within the protein and inaccessible. Weak biotinylation was seen for the N184C and S194C mutants. At A182C, F185C, T186C, F188C, K190C, S192C and F195C mutants robust MTSEA-biotinylation was seen with apyrase and this was reduced or abolished following ATP pre-treatment suggesting that the accessibility of these residues is blocked either directly by ATP binding or as a result of a conformational change in the receptor. MTSEA-biotinylation was detected for P196C-V200C mutants in the presence of apyrase or ATP indicating that these residues are accessible in either the resting or activated conformation of the P2X1 receptor. The pattern of biotinylation between Thr186 and Ser194 with alternative residues being biotinylated is consistent with a beta sheet and substantiates bioinformatic predictions.

## Discussion

In this study, we used cysteine scanning mutagenesis of Glu181 to Val200 to provide an unbiased insight into the contribution of this region to P2X1 receptor properties. The majority of the mutants had no effect on either ATP potency or MTS reagent action and can be discounted as essential for normal receptor function and aid in the refinement of models of residues important in mediating ATP action. The study highlights a significant contribution of residues Phe185, Thr186, Phe188, Lys190 and Phe195 in mediating the actions of ATP and gives insight into the surface accessibility and conformational changes in the receptor on activation.

The Phe185Thr186 doublet is conserved throughout the P2X receptor family (the exception being a conservative substitution of a tyrosine for phenylalanine in the Schistosome P2X receptor) (Agboh *et al.* 2004). The lack of effect of MTS reagents at the F185C mutant even though they are accessible to MTSEA-biotinylation confirms and extends previous studies on P2X2 and P2X4 receptors (Roberts *et al.* 2008) and suggest that this residue does not face directly into the ATP binding pocket. However the reduction in partial agonist responses for the F185C mutant suggests that this may result from a localized effect on the adjacent threonine residue. The mutation T186C at the P2X1 receptor gave a modest ∼ 8-fold decrease in ATP potency similar to that for the T186A mutant and equivalent cysteine mutants at P2X2 and P2X4 receptors (Roberts *et al.* 2008) in addition the partial agonist actions of BzATP and Ap_5_A were essentially abolished. We used 2-azido ATP binding to address directly whether changes in agonist potency result from an effect on the initial binding step (that may involve conformational change) or the subsequent activation of the channel (gating). At the T186C mutant ATP potency and 2-azido ATP binding was reduced under control conditions compared to WT and ATP sensitivity and 2-azido ATP binding was further reduced by the MTS reagents. In contrast at F195C agonist action was reduced by MTS reagents with no effect on 2-azido ATP. These results suggest that the 2-azido ATP assay can be used to discriminate mutations and treatment that acts on the agonist binding step (T186C) from effects on the subsequent opening of the channel (F195C). The sensitivity of the T186C mutant to MTS reagents was independent of the charge and appeared to result from the bulk of the substitution like that reported for the P2X4 receptor (Roberts *et al.* 2008). This suggests that this residue is not involved in coordination of the charged phosphates of ATP and is likely to be involved in binding of the adenine or ribose groups.

The ATP sensitivity of the K190C mutant showed a modest 3–4 fold decrease compared to WT channels. ATP potency was increased back to WT levels by treatment with positively charged MTSEA and inhibited by negatively charged MTSES demonstrating the contribution of charge at this position to agonist action. This is the same as the charge dependence of MTS reagent effects at the cysteine mutants of the conserved lysine residues implicated in ATP binding at the receptor (Roberts and Evans 2007; Roberts *et al.* 2008). Lys190 is conserved in all the human P2X receptors as well as the Schistosome P2X (Agboh *et al.* 2004) with a conservative Arg substitution at the Algal P2X receptor (Fountain *et al.* 2008). Interestingly the equivalent residue in the Dictyostelium P2X receptor is a negatively charged aspartate (Fountain and North 2006). The reduction in ATP sensitivity on MTSES treatment at the K190C mimics this negative charge and decreased ATP potency and suggests that the reduced ATP potency at the Dictyostelium receptor may result in part from this charge difference. The reduction in 2-azido ATP cross-linking at K190C by MTSES, and decreased efficacy of the partial agonists suggests that the change in potency results from a decrease in agonist binding. The modest effects of the Lys190 mutation suggest that this residue may function to fine tune agonist sensitivity at P2X1 receptors and this residue is not as important in mediating the action of ATP as conserved Lys68 and Lys308. However more dramatic decreases in potency have been reported for the alanine mutations of the equivalent lysine residues at P2X2 and P2X4 receptors (200 and 2000-fold respectively) (Ennion *et al.* 2000; Jiang *et al.* 2000; Yan *et al.* 2005) indicating that at these receptors the lysine makes a greater contribution. This could be as in these receptors the lysine is perhaps closer to the binding pocket. This is supported by recent studies from P2X2–4 receptors suggesting that there may be some differences in the dimensions of the ATP binding pocket between receptor subunits (Marquez-Klaka *et al.* 2009). Alternatively the conserved lysine could play a subtype dependent role in interactions with other charged residues in the extracellular loop.

An aromatic residue is conserved at position 195 (P2X1 receptor numbering) throughout the mammalian, Schistosome and Algal P2X receptors and in the Dictyostelium receptors there is an adjacent tyrosine residue. Responses were essentially abolished by treatment of F195C mutants with MTS reagents (even to a supra-maximal concentration of ATP) and following MTS treatment the level of azido-ATP binding was unaffected. This demonstrates that the reduction in response at F195C does not result from a decrease in agonist binding to the receptor but from an effect on either gating and/or ionic permeation. The reduction in MTSEA-biotinylation at the F195C mutant in response to ATP demonstrates a conformation change in the receptor on activation rendering the position inaccessible. One interpretation of these results is that the incorporation of the MTS reagents inhibits the conformational change so stopping gating of the channel. Interestingly the adjacent region Pro196 to Val200 can be MTSEA-biotinylated (and R197C shows a decrease in partial agonist action suggesting that this region may have some minor effect on channel properties). However, the sensitivity of Pro196 to Val200 to MTSEA-biotinylation this is unaffected by ATP application, suggesting that Phe195 could act as a pivot around which conformational changes occur.

The results from the present study show that the region Phe185-Phe195 plays an important role in channel regulation. In the stretch Thr186-Ser192 alternative residues were MTSEA-biotinylated. This is consistent with bioinformatic predictions that this region of the receptor forms a beta sheet (Digby *et al.* 2005). Similarly Thr186, Phe188 and Lys190 mutants showed decreases in ATP potency and partial agonist action consistent with a beta sheet. The lack of effect of MTSEA or MTSES at F188C suggests that this residue does not make as close contact to the ATP molecule as Thr186 and Lys190. We therefore tested the larger MTS reagent [2-(Trimethylammonium) ethyl] methanethiosulfonate Bromide) (MTSET). MTSET (1 mM) had little or no effect on WT P2X1 receptor mediated currents (115 ± 6.5% of control). At the F188C mutant MTSET (1 mM) reduced the response to an EC_50_ concentration of ATP (5 μM) by 76.6 ± 6.6% (*p* < 0.001, *n* = 4). This reduction in amplitude was also accompanied with a slowing in the time-course of the ATP current consistent with a reduction in agonist potency and consistent with a contribution to the agonist binding pocket. Taken together these results suggest that Thr186, Phe188 and Lys190 face the ATP binding pocket. The biotinylation of the face of the beta sheet was also sensitive to the application of ATP indicating that the accessibility of the beta sheet is either blocked by ATP binding (Thr186, Phe188 and Lys190) or closure of the agonist binding pocket. At either end of the beta sheet are conserved phenylalanine residues that we have suggested contribute to conformational changes (through effects on partial agonists F185C or MTS action F195C) in response to agonist action.

**Fig. 7:**
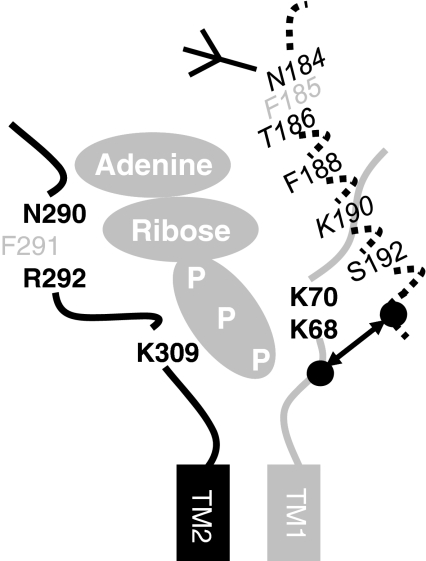
Model of the ATP binding site. Portions of the extracellular loop adjacent to either the first (in gray) or second (in black) transmembrane segment are shown for two adjacent P2X1 receptor subunits. Conserved residues that have been shown to contribute to ATP potency are shown (gray residues correspond to conserved aromatic residues in areas of high conservation). The dotted line corresponds to the region Glu181-Val200 that was characterized in the present study, the zig-zag line corresponds to the region of beta sheet as indicated by the pattern of MTSEA-biotinylation. Based on studies on the zinc and copper binding site of P2X7 receptors (Liu *et al.* 2007) it is possible that residues His62 and Asp197 (P2X7 receptor numbering, indicated by black balls) are close together (shown by arrow) as suggested for residues involved in the zinc binding site at P2X2 receptors (Nagaya *et al.* 2005). This would bring the residues Phe195, Lys190 and Thr186 close to the putative ATP binding site. If this is the case Lys190 could be close to the other conserved lysine residues and this could account for the charge dependent effects of MTS reagents on the K190C mutant. Asn184 is glycosylated (Roberts and Evans 2006).

The region Phe185-Phe195 is in the middle of the extracellular loop sequence ∼ 95–116 residues from others suggested to be in the ATP binding pocket. A model for P2X1,2&4 receptors suggests that the lysines Lys68,70&309 along with Asn290Phe291Arg292 (P2X1 receptor numbering) co-ordinate ATP binding (Roberts *et al.* 2008) and the phenylalanine residue is close to the first lysine (Marquez-Klaka *et al.* 2007, 2009). How could the region Thr186-Phe195 interact with this proposed binding pocket? Recent mutagenesis studies on the P2X7 receptor showed that His62 (two residues before the conserved lysine doublet equivalent to Lys68 and Lys70 in the P2X1 receptor) and Asp197 (equivalent to Ser194 in the P2X1 receptor) contribute to zinc and copper inhibition (Liu *et al.* 2007). This raises the possibility that these residues could be in close proximity as demonstrated for two histidine residues in adjacent subunits of the P2X2 receptor that are essential for zinc potentiation (Nagaya *et al.* 2005). Such an interaction would bring the region Thr186-Phe195 close to the predicted ATP binding site, provide a contribution of Lys190 to the ATP binding pocket, and a role of Phe195 in gating/ionic permeation (Fig. 7).

In summary analysis of mutants in the region Glu191-Val200 has highlighted the contribution of a beta sheet region of the P2X1 receptor to ATP action and provided an updated model of the agonist binding site. The work also shows that the residue Phe195 in the middle of the extracellular loop plays a role in channel gating and suggests that there are widespread changes in the conformation of the receptor on activation.

## Abbreviations used

Ap_5_A: P^1^,P^5^-diadenosine 5′-pentaphosphate
BzATP: 2′,3′-*O*-(4-benzoyl)-ATP
MTS: methanethiosulfonate
MTSEA: (2-aminoethyl)methanethiosulfonate hydrobromide
MTSES: sodium (2-sulfonatoethyl) methanethiosulfonate
MTSET: [2-(Trimethylammonium) ethyl] methanethiosulfonate Bromide)
WT: wild type
